# Rope skipping or badminton? exercise reduced sleep onset latency in university students

**DOI:** 10.3389/fspor.2025.1514596

**Published:** 2025-05-22

**Authors:** Zixin Ye, Shuyue Tan, Yingyuan Zhu, Jialin Fan

**Affiliations:** ^1^ School of Psychology, Shenzhen University, Shenzhen, Guangdong, China; ^2^ The Shenzhen Humanities & Social Sciences Key Research Bases of the Center for Mental Health, Shenzhen, Guangdong, China

**Keywords:** exercise intervention, aerobic exercise, PSQI, Chinese university student, daytime dysfunction

## Abstract

**Introduction:**

Poor sleep quality is common among university students and can negatively affect their physical and mental health. Aerobic exercise has shown promise in mitigating these issues. Exercise interventions involved in previous studies were often time-consuming. To identify a more efficient approach, we evaluated the effects of 13-day rope skipping and badminton interventions on sleep issues. We hypothesized that both badminton and rope skipping could effectivly improve sleep problems and that the effect of rope skipping would be greater than that of badminton.

**Methods:**

Fifty-five participants’ levels of sleep were assessed via nine variables, using the Consensus Sleep Diary and the Pittsburg Sleep Quality Index.

**Results:**

Both exercises were effective in improving sleep onset latency. Badminton has a greater effect on improving daytime dysfunction than rope skipping. No additional significant results were found on the other components of sleep. One of the reasons for the nonsignificant results could be that some participants’ sleep was disturbed by their roommates sharing the same bedroom.

**Discussion:**

Short-term rope skipping and badminton are competitive interventions in improving sleep quality for university students. Some participants reported being more interested in exercise after the intervention, suggesting that exercise interventions could be used to foster exercise habits.

## 1 Introduction

Sleep plays a vital role in individuals' quality of life, working efficiency, and creativity (1, 2). Sleep problems can be caused by high levels of stress, anxiety depression, and poor psychological well-being (3–5). Among Chinese university students, this can also be attributed to academic workload and employment concerns (6, 7). Previous studies have shown that exercise interventions can be beneficial for sleep issues (8–10). However, the types of exercises used in these studies were often unspecified or required a significant time investment [e.g., (6–8, 10)], which might not be efficient for university schedules. Thus far, a type of exercise intervention that is more suitable for Chinese university students has not been identified.

Sleep problems are common among Chinese university students (6, 7). Due to heavy academic workload and concerns about high unemployment rates for recent graduates, 43.03% of university students reported not getting a good night's sleep (6, 7, 11, 12). Students who perceive poor sleep quality are likely to have poor academic performance and be dissatisfied with their academic performance (13, 14). As a result of poor sleep quality, students may also suffer from higher levels of anxiety, depression, poor attention, and memory loss (15, 16). Further, the risk of common psychiatric disorders may significantly increase (15).

The effect of physical exercise on sleep disturbances has been widely studied in recent years, and the results have been mixed. While some researchers have failed to find convincing evidence of its effectiveness (17), the potential of exercise cannot be disregarded. Indirect evidence supports the value of exercise interventions substantiated for reducing the incidence of daytime sleepiness and cognitive dysfunction caused by sleep deprivation (18). Additionally, exercise as medicine is highly effective in treating insomnia and alleviating sleep problems and stress in clinical psychiatric disorders, such as depression (19–22). Similar evidence was also provided in other studies that engaging in aerobic exercises can lead to improved sleep quality after several weeks of consistent or intermittent exercise (8, 23, 24). Furthermore, increasing the intensity of the exercise from mild (approximately 45∼50% of maximum heart rate) to moderate (approximately 65∼70% of maximum heart rate) can enhance these effects (10).

In past studies of exercise interventions, researchers typically compared the effects of aerobic or anaerobic exercise with a control group [e.g., (6, 7, 10, 23)] or with participants receiving other interventions, such as meditation (8). However, the efficiencies among different exercise interventions have never been compared. Moreover, most experimental procedures ranged from two to 12 months (24). Given the heavy academic workload of Chinese university students, it is likely that they have limited spare time for long-term aerobic exercises. Therefore, to relieve fatigue and sleep problems in Chinese university students more efficiently, it is necessary to identify a type of aerobic exercise that is more convenient and time-saving than existing options without compromising effectiveness.

In China, rope skipping and badminton are popular among university students because they require fewer motor skills, fields, and equipment than other sports (25, 26). Li et al. (6, 7), found that a short-term (six consecutive days) physical exercise at moderate intensity could improve sleep quality effectively. Hence, our aim was to investigate the effects of a similarly short-term rope skipping or badminton exercise on improving sleep quality. We hypothesized that both rope skipping and badminton exercises would improve sleep quality among the participants. We also hypothesized that the effect of rope skipping would be greater than that of badminton because badminton requires cooperation between two players, which may demand additional mental energy.

## 2 Method

### 2.1 Participants

Seventy-eight university students were recruited for the present study. Students who met the following criteria were included: (1) had no prior exercise habits; (2) reported a Pittsburgh Sleep Quality Index (PSQI) score higher than seven, indicating poor sleep quality (27); (3) had no diseases or hypoglycemic symptoms; (4) were not taking any medication; and (5) were not in their menstrual period if they were female.

Due to the limited number of sports equipment, the present study was conducted in three stages. We initiated the recruitment procedure one month prior to each stage. Once at least 15 individuals met the inclusion criteria, we commenced the current stage and assigned the participants to the experiment, resulting in 24 to 28 participants for each stage and a total of 78 participants included in the study. The participants were assigned to one of three groups (the badminton group, the rope skipping group, or the control group) based on their self-reported preferences, which were collected through interviews. All participants were instructed to avoid consuming alcohol, caffeinated drinks, tobacco, and any sleep aids throughout the experiment. In addition, all participants were asked to maintain their regular daily schedules and were advised to go to bed before 12 a.m. During the experiment, 9 participants dropped out, 6 participants lacked testing, 6 participants were unwell, and 2 participants had irregular daily schedules. After excluding these participants, we analyzed data from 55 participants (45 females and 10 males) aged between 18 and 28 years. Of these participants, 19 were in the control group, 19 were in the badminton group, and 17 were in the rope skipping group.

### 2.2 Materials

#### 2.2.1 Sleep measurement

##### 2.2.1.1 Consensus sleep diary

The Consensus Sleep Diary was developed for the purposes of assessing sleep variables such as perceived sleep onset latency (SOL), wake after sleep onset, and sleep quality (28). In the original article, the diary was published in three versions: the Core Consensus Sleep Diary, the Expanded Consensus Sleep Diary for Morning, and the Expanded Consensus Sleep Diary for Evening (28).

The questions included in the present study were extracted from the Core Consensus Sleep Diary, which originally consisted of nine questions (Supplementary Table S1). We excluded question 5 to avoid the possible excessive burden. Additionally, we combined questions 1 and 2 and rephrased them to read “What time did you turn off the light and go to bed?” and rephrased question 3 to read “How long did it take you to fall asleep after getting into bed?”. The reason for these revisions was that all participants slept in dormitories, where students typically go to bed and simultaneously go to sleep when the main lights are turned off by dormitory managers at a designated time at night.

Furthermore, we revised the response options for question 8 to a Likert scale from zero to 10, with 0 = extremely poor and 10 = extremely good. We also added a question for the serial number of the participants. We abandoned question 9 in the original core diary because the participants were informed that they could contact the experimenters at any time. Finally, we translated all the questions into Chinese. All participants were required to complete their sleep diaries every morning after waking up throughout the experiment.

##### 2.2.1.2 Pittsburgh sleep quality Index (PSQI)

The Pittsburgh Sleep Quality Index (PSQI), developed by Buysse et al., is a widely used measurement for assessing sleep (29). It comprises 19 items evaluating 7 components of sleep, including subjective sleep quality, sleep latency, sleep duration, sleep efficiency, sleep disturbances, the use of sleeping medication, and daytime dysfunction. Each component is scored on a scale of zero to three points, with a maximum global PSQI score of 21. Higher scores indicate poorer sleep quality (29). For the current study, we used the Chinese version of the PSQI, translated by Liu et al. (27). Cronbach's alpha was 0.799 in the present study, indicating good reliability. All participants were instructed to complete the PSQI before and after the experiment.

#### 2.2.2 Sports equipment

For the badminton group, we provided six identical Decathlon Perfly badminton rackets and more than a dozen DHS 402 badminton shuttlecocks. For the rope skipping group, we provided 30 identical Decathlon skipping ropes. Due to the limited availability of equipment, participants were instructed to share the equipment and take turns using it to ensure that everyone had equal access to the equipment.

### 2.3 Procedure

**Figure 1:**
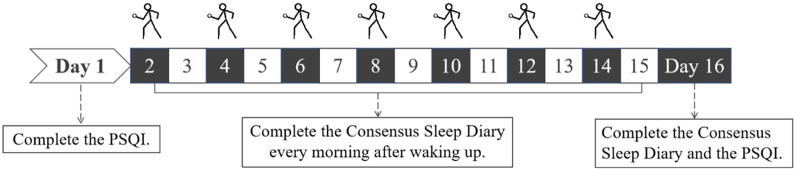
The processure of the intervention.

The present study was conducted in three stages, with each participant undergoing the experiment for 16 days. The procedure of the intervention is shown in Figure 1. On the first day of each stage, all participants completed the PSQI. The participants were instructed to maintain daily routines as usual, and report if sudden changes occur during the intervention period that could possibely affect sleep. They were also informed that they could contact the experimenters at any time if they had any questions or concerns. Starting from the first day of each stage, participants in the badminton group and the rope skipping group arrived at a designated site on alternate days (i.e., the 2nd, 4th, 6th, 8th, 10th, 12th, and 14th days of each stage) between 5 p.m. and 7 p.m. Following a 2–5 min warm-up exercise, they performed their assigned exercise for 30 min, after which they completed a short questionnaire to record their exercise type and total exercise time. Additional exercise was not allowed for either group for the duration of the experiment.

The participants were instructed to maintain an intensity level that, compared to their resting state, induced noticeably heavier breathing and sweating during the 30 min exercise periods, while avoiding extreme fatigue, gasping for breath, or muscle exhaustion. All exercise sessions were conducted indoors on standard PVC sports flooring, within an area measuring 13.4 m × 6.1 m—the official dimensions regulated by the Badminton World Federation. Participants in the badminton group were also allowed to play on standard badminton courts when available. All participants were instructed to focus solely on maintaining the required intensity level during the exercise sessions, rather than on technical performance. Specifically, on one hand, participants in the rope skipping group were not required to perform strictly consecutive jumps or use advanced rope skipping techniques. A reasonable number of failed attempts was allowed—as long as participants did not fail on every jump. After a failed attempt, they were instructed to resume skipping within 30 s, without taking extended breaks. On the other hand, participants in the badminton group were not required to perform consecutive rallies throughout the entire session or use advanced hitting techniques. However, they were instructed to avoid frequent missed hits and to maintain continuous movement. Extended breaks longer than 30 s were not permitted.

Meanwhile, the control group was instructed to maintain their regular daily activities and refrain from engaging in any sports. Furthermore, all participants completed the Consensus Sleep Diary every morning after waking up. On the 16th day of each stage, all participants completed the PSQI once again. The data were then checked and imported.

### 2.4 Data analysis

The data from the Consensus Sleep Diary and PSQI were analyzed using IBM SPSS version 23. The statistically significant alpha level was set to be less than 0.05. Mixed analysis of variance (ANOVA) (Repeated measures, within-between interaction) tests were conducted for two purposes: to assess the effects of both badminton and rope skipping exercises on the participants' sleep quality, and to assess the differences between them.

## 3 Results

The present study aimed to analyze SOL and PSQI scores. SOL was assessed based on data obtained from the Consensus Sleep Diary and PSQI. The PSQI scores were analyzed in their entirety. PSQI scores were evaluated based on seven items from the PSQI (including sleep quality, sleep latency, sleep duration, sleep efficiency, sleep disturbances, the use of sleeping medication, and daytime dysfunction) and the global PSQI score. Hence, the present study included the assessment of nine variables.

A total of 18 Mixed ANOVA tests, consisting of two tests for each of the nine variables, were conducted to examine the effects of badminton and rope skipping. In the first ANOVA test for each variable, the badminton group and the rope skipping group were combined and labelled as the “exercise groups”, which were subsequently compared to the control group. The second ANOVA test for each variable focused specifically on the difference between the badminton group and the rope-skipping group.

### 3.1 SOL

The SOL data (from the Consensus Sleep Diary) from the first day at all stages were excluded due to the first night effect. The first-night effect refers to the phenomenon that participants often experience poorer sleep quality during initial sleep monitoring due to the inability to acclimatise to the sleep-monitoring environment (30). As a result, only 14 days of SOL data (from the Consensus Sleep Diary) were used for each participant. In addition, Only 48 participants completed the PSQI before and after the intervention, of which 18 were in the control group, 17 in the rope skipping group, and 13 in the badminton group.

**Figure 2:**
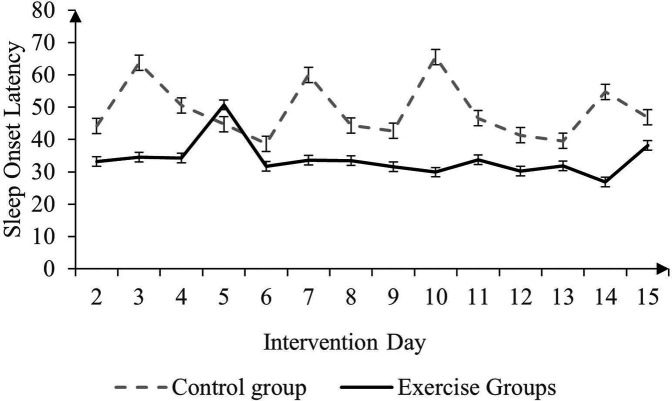
Comparison of mean SOL (in minutes) of the exercise groups and control group over time.

**Figure 3:**
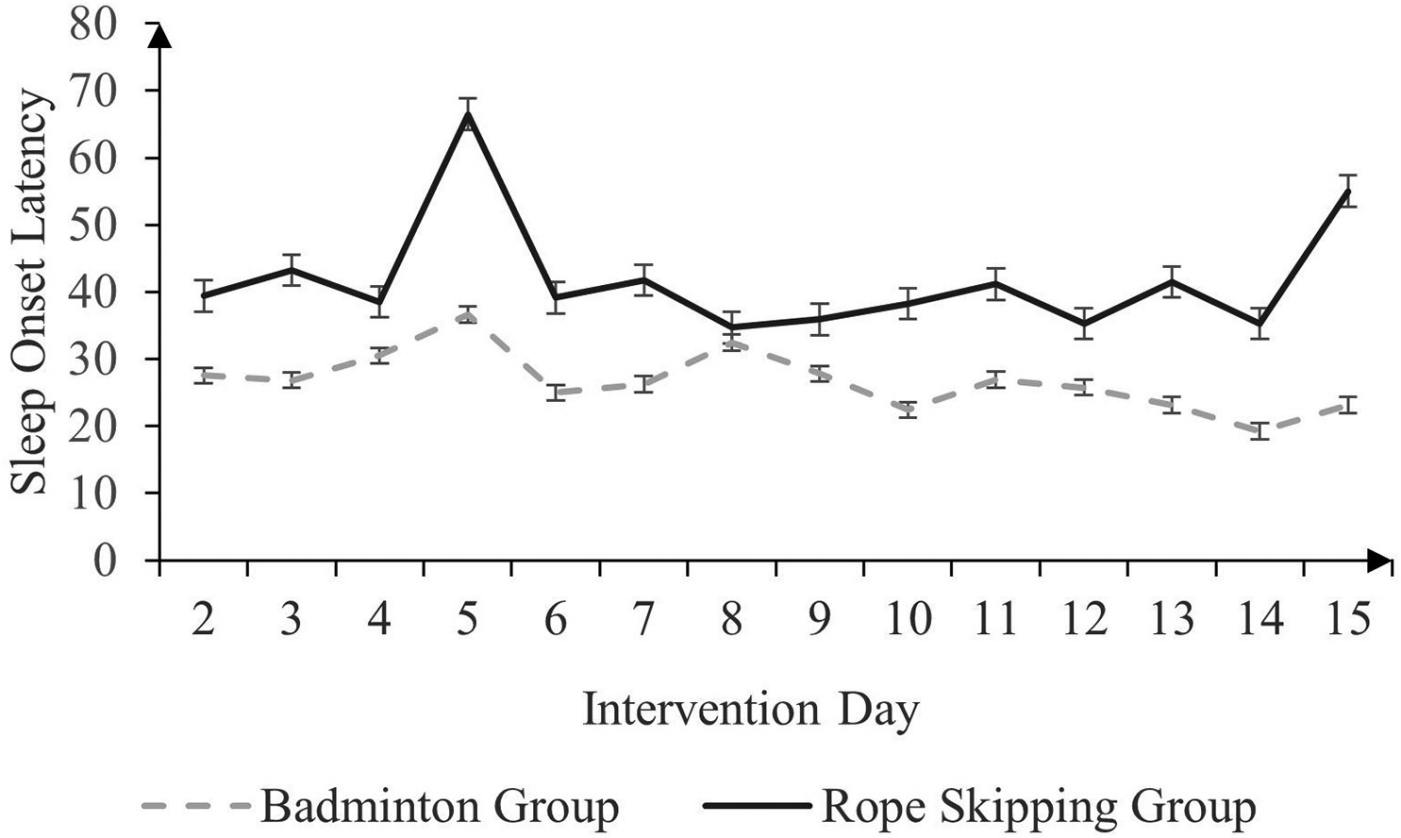
Comparison of mean SOL (in minutes) of the rope-skipping group and badminton group over time.

The main effect of time on SOL (The Consensus Sleep Diary) was found to be significant in only test one (Table 1), F (12, 53) = 1.938, *p* = 0.05, *η^2^* = 0.035. The average SOL (The Consensus Sleep Diary) of the exercise groups (M = 33.837, SD = 28.071) was significantly lower than that of the control group (M = 48.816, SD = 40.852), F (1, 53) = 5.322, *p* < 0.05, *η*^2^ = 0.091. The average SOL (The Consensus Sleep Diary) of the badminton group (*M* = 26.688, *SD* = 21.973) was significantly lower than that of the rope skipping group (*M* = 41.828, *SD* = 31.751), F (1, 34) = 8.261, *p* < 0.01, *η^2^* = 0.195. Significant interaction effects were detected only between the exercise groups and the control group, F (12, 54) = 2.502, *p* = 0.01, *η^2^* = 0.045 (Figures 2,3). The main effect of time on SOL (PSQI) was found to be significant only in test one (Table 2), F (1, 46) = 10.724, *p* < 0.01, *η^2^* = 0.189.

**Table 1:** Mixed ANOVA for SOL of the exercise groups and the control group.

Variables		*MS*	*F*	*p*	*η^2^*
SOL (The Consensus Sleep Diary)	Time	1844.698	1.938	0.050*	0.035
	Group	39062.262	5.322	0.025*	0.091
	Group × Time	2381.377	2.502	0.010*	0.045
SOL (PSQI)	Time	3.117	10.724	0.002**	0.189
	Group	1.056	1.289	0.262	0.027
	Group × Time	0.117	0.404	0.528	0.009

* *p* < .05.

** *p* < .01.

MS, mean square.

**Table 2:** Mixed ANOVA for SOL of the rope-skipping group and the badminton group.

Variables		*MS*	*F*	*p*	*η^2^*
SOL (The Consensus Sleep Diary)	Time	3043.739	2.231	0.055	0.062
	Group	28791.620	8.261	0.007**	0.195
	Group × Time	1491.307	1.093	0.366	0.031
SOL (PSQI)	Time	1.255	3.481	0.073	0.111
	Group	0.769	0.983	0.330	0.034
	Group × Time	0.055	0.152	0.699	0.005

MS, mean square.

** *p* < .01.

### 3.2 Other dimensions of sleep

The pre-intervention PSQI scores of all participants were significantly higher than their post-intervention scores, F (1, 46) = 28.362, *p* < 0.001, *η^2^* = 0.381, F (1, 30) = 21.454, *p* < 0.001, *η^2^* = 0.434. Additionally, the PSQI scores of the exercise groups differed significantly from those of the control group, F (1, 46) = 6.185, *p* < 0.05, *η^2^* = 0.119. However, no significant interaction effects were found (Supplementary Tables S2, S3), indicating that the changes in global PSQI scores before and after the intervention did not significantly differ between groups.

The main effect of time on sleep quality was found to be significant in both test one (Supplementary Table S2), F (1, 46) = 20.674, *p* < 0.001, *η^2^* = 0.310, and test two (Supplementary Table S3), F (1, 1. 30) = 21.157, *p* < 0.001, *η^2^* = 0.430. The pre-intervention Sleep efficiency (PSQI scores) of all participants were significantly higher than their post-intervention scores, F (1, 46) = 4.488, *p* < 0.05, *η^2^* = 0.089. The average Sleep duration (PSQI scores) of the rope skipping group (*M* = 0.38, *SD* = 0.61) was significantly lower than that of the badminton group (*M* = 0.57, *SD* = 0.64), F (1, 30) = 5.305, *p* < 0.05, *η^2^* = 0.159. The pre-intervention Sleep disturbances (PSQI scores) of the exercise groups participants were significantly higher than their post-intervention scores, F (1, 30) = 7.251, *p* < 0.05, *η^2^* = 0.206 (Supplementary Table S3, S4). Significant interaction effects were detected only between the rope skipping group and the badminton group for daytime dysfunction, F (1, 30) = 6.378, *p* < 0.05, *η^2^* = 0.186 (Supplementary Tables S2, S3). There was a marginally significant difference in daytime dysfunction between the exercise groups and the control group, F (1, 46) = 3.208, *p* = 0.080 (Supplementary Table S2). The means and standard deviations of all variables are shown in Table 3 and Supplementary Table S4.

**Table 3:** Comparisons of components of sleep of the exercise groups and the control group.

Variables		*M* *±* *SD* (exercise groups)	*M* *±* *SD* (control group)
Sleep quality	before	1.667 ± 0.102	1.722 ± 0.131
	after	1.167 ± 0.101	1.333 ± 0.131
Sleep duration	before	0.67 ± 0.11	0.56 ± 0.14
	after	0.47 ± 0.11	0.44 ± 0.14
Sleep efficiency	before	0.53 ± 0.14	0.78 ± 0.18
	after	0.33 ± 0.12	0.44 ± 0.16
Sleep disturbances	before	1.17 ± 0.09	1.28 ± 0.12
	after	0.90 ± 0.09	1.22 ± 0.12
The use of sleeping medication	before	0.10 ± 0.08	0.22 ± 0.11
	after	0.10 ± 0.07	0.17 ± 0.09
Daytime dysfunction	before	2.37 ± 0.12	2.39 ± 0.15
	after	1.63 ± 0.12	2.11 ± 0.16
Global PSQI score	before	8.43 ± 0.29	9.17 ± 0.38
	after	6.23 ± 0.37	7.50 ± 0.47

## 4 Discussion

The present study aimed to investigate and compare the effects of short-term exercise interventions of badminton and rope skipping on sleep problems among Chinese university students with poor sleep quality (PSQI score > 7) and without any exercise habits. Fifty-five participants were divided into three groups: a badminton group, a rope-skipping group, and a control group. A 13-day exercise intervention was assigned to the badminton and rope skipping groups. Our first hypothesis was partially supported—both exercises were effective only in improving sleep onset latency and daytime dysfunction—while the second hypothesis was rejected, as badminton has a greater effect on improving daytime dysfunction than rope skipping.

Performing exercise led to a shortened sleep onset latency, as participants who engaged in physical activity during the study spent less time falling asleep, while those in the control group did not. Similar findings were reported by Kalak et al. (23), who found that adolescents participating in regular physical activity experienced significant improvements in sleep onset latency as measured by EEG recordings. A systematic review by Chennaoui et al. (31) also noted that moderate-intensity aerobic exercise before bedtime can reduce sleep onset latency, as supported by the findings of Passos, D'Aurea, et al. (32) and Passos, Garbuio, et al. (33).

The current study did not observe significant changes in global PSQI scores, although prior research has consistently supported the beneficial effects of exercise on PSQI outcomes (21, 31). However, the sleep disturbance subscale of the PSQI showed significant pre-post improvements within the exercise groups, whereas no statistically significant differences were observed between the badminton and rope skipping groups. Several factors may explain this result. First, the intervention was relatively short and low in frequency (alternate-day sessions over 13 days), which may not have been sufficient to produce group-level differences in sleep disturbances. Second, the relatively small sample size may have limited the statistical power to detect subtle changes across conditions. Lastly, it needs to be noted that the cut-off point criteria for the PSQI applied in the present study is different than the more usually accepted criteria. Liu et al.'s study found that a cut-off point of 7 scores is more appropriate for Chinese than the original cut-off point (5 scores) (27). However, the study by Liu et al. was conducted in 1996. Therefore, the data and criteria could be outdated. Another Chinese PSQI validation study is needed in the future to update the Chinese PSQI cut-off point.

A growing body of evidence showed that exercise improves daytime dysfunction among adolescents and adults (8, 18). For instance, exercise helps to improve mental health and thus indirectly improves daytime dysfunction (34). Those who regularly engage in physical exercise were less likely to have daytime sleepiness than those who did not (35). The results of our study are consistent with previous research. We further found that badminton was more effective than rope skipping in improving daytime dysfunction, which might imply that additional mental energy was not demanded in two-player sports such as badminton, contrary to our hypothesis. Interaction during exercise may be the reason for the differences in daytime dysfunction between badminton and rope skipping. Social exercise has been associated with enhanced psychological benefits compared to solo exercise (36).

Compared to rope skipping, badminton is a two-player sport that inherently involves social engagement and communication. In the present study, some participants reported gaining positive social interactions with other participants–that is, for instance, they developed friendships with their partners. Whereas participants in the rope-skipping group completed the experiment in isolation. No additional significant results were found on the other components of sleep between badminton and rope skipping. However, it should be noted that participants were grouped based on their inclination. Additional differences may be identified if randomization is utilized in future studies. Therefore, while it may imply that doubles sports are more relaxing than individual sports, further research is necessary to assess the comparability of different exercises and sports. It is also important for future intervention studies to attempt to flexibly combine a variety of exercise and sports.

We extended the existing findings on short-term exercise interventions and corroborated that a 13-day intervention with exercising on alternate days can improve SOL in university students who do not include exercise in their daily routines. By comparing the two sports, we also found that badminton improved daytime dysfunction more than rope skipping. Compared to strictly standardized laboratory studies, our findings are more representative of real-life scenarios.

Moreover, it is worth noting that 44.4% of the participants in the current study reported a willingness to continue exercising even after the experiment ended. This suggests that short-term exercise interventions are likely to enhance individuals' interest in exercise. Exercise might not be a sustainable strategy for addressing sleep problems in terms of solving such problems. However, it is sustainable in terms of giving college students in developing countries a go-to long-term solution to coop acute insomnia and daytime sleepiness. We recommend that university students, or individuals who have limited spare time, perform rope skipping or badminton on alternate days for 13 days if they drowsiness during the day or struggle to fall asleep. For future studies, follow-up surveys are encouraged to investigate whether participants developed regular exercise habits. Furthermore, in light of these findings, clinical practitioners can incorporate badminton and rope skipping exercises as quick-acting interventions for individuals suffering from psychiatric disorders because these short-term exercises may alleviate symptoms by mitigating sleep issues. If exercise is included as a part of the intervention plan but clients refuse to comply, these two exercises can also serve as strategies to encourage and establish daily exercise routines. However, further evidence is required to substantiate the potential benefits of badminton and rope skipping in clinical practises.

### 4.1 Limitations

The present study has several limitations that should be considered when interpreting the results. As mentioned earlier, some groups, such as the badminton group during the analysis of PSQI scores, had small sample sizes, which could reduce the statistical power and effect size of the results and could contribute to the insignificant results of the PSQI scores. In addition, the attrition rates in the present study were relatively high. The present study originally planned to use Actigraphy as an objective sleep measurement tool, but due to the incorrect use of the actigraphs by some participants, all the data from actigraphy being discarded. Moreover, participants completed the required questionnaires on time during the experiment, whereas some failed to complete or completed the post-experiment PSQI test at random times. This might suggest that they perceived the end of the experiment as less important than the experiment itself. Both questionnaires and actigraphy require participants to comply with the instructions. To address this issue in future studies, researchers should consider arranging rehearsals or practice sessions before the start of the experiment, supervising participants to complete the questionnaire on time during the experiment, and emphasising the importance of the posttest task to participants.

Another limitation of this study is that we failed to exclude a potential confounder: the dormitory environment. Some participants reported concerns about this factor, which has been shown to affect sleep quality among university students (37). In Chinese university dormitories, students usually share the same bedroom with one to five other roommates. Participants in the present study reported that maintaining ideal sleep quality was challenging when their sleep was easily disturbed by roommates and that their sleep quality often improved when roommates were away on weekends. On one hand, the dormitory environment is likely to be a significant confounding factor that affects the results relevant to sleep; therefore, researchers should take it into consideration for future sleep studies conducted in Chinese universities. On the other hand, in future research on sleep issues, the effects of interventions could be evaluated for university students whose sleep quality is severely disturbed by external factors.

## 5 Conclusion

This study examined the effects of a short-term badminton and rope-skipping intervention, conducted on alternate days over a 13-day period, on sleep quality among Chinese university students with poor sleep and no regular exercise habits. The findings suggest that both forms of exercise effectively reduced sleep onset latency, with badminton showing a greater benefit than rope skipping in alleviating daytime dysfunction. These results highlight the potential of short-term physical activity, even when performed intermittently, to address specific dimensions of sleep problems in young adults. Furthermore, the observation that nearly half of the participants expressed a willingness to continue exercising after the study indicates that such interventions may also help foster long-term exercise habits.

Based on these findings, we recommend that university students—especially those with limited time or irregular schedules—consider engaging in short-term aerobic activities such as rope skipping or badminton to mitigate sleep difficulties. Given the low cost, accessibility, and psychological benefits of these exercises, this approach may also be relevant across cultural contexts and could serve as a practical non-pharmacological strategy to improve sleep quality globally.

## Data Availability

The raw data supporting the conclusions of this article will be made available by the authors, without undue reservation.
