# Supplementary material for: Rope skipping or badminton? exercise reduced sleep onset latency in university students
Source: Front Sports Act Living. 2025 May 22;7:1514596. doi: 10.3389/fspor.2025.1514596 (PMC12137340; doi:10.3389/fspor.2025.1514596)
Supplement: Supplementary file 1 [file Table1.docx]

Supplementary Material

# The Core Consensus Sleep Diary

1. **The Core Consensus Sleep Diary in English:**

| 1. What time did you get in to bed?  Write the time that you got into bed. This may not be the time that you began “trying” to fall asleep. |
| --- |
| 2. What time did you try to go to bed?  Record the time that you began “trying” to fall asleep.. |
| 3. How long did it take you to fall asleep?  Beginning at the time you wrote in question 2, how long did it take you to fall asleep.  ________ minutes |
| 4. How many times did you wake up, not counting your final awakening?  How many times did you wake up between the time you first fell asleep and your final awakening?  ________ times |
| 5. In total, how long did these awakenings last?  What was the total time you were awake between the time you first fell asleep and your final awakening. For example, if you woke 3 times for 20 minutes, 35 minutes, and 15 minutes, add them all up (20+35+15= 70 min or 1 hr and 10 min).  ________ hour ________minutes |
| 6. What time was your final awakening?  Record the last time you woke up in the morning. |
| 7. What time did you get out of bed for the day?  What time did you get out of bed with no further attempt at sleeping? This may be different from your final awakening time (e.g. you may have woken up at 6:35 a.m. but did not get out of bed to start your day until 7:20 a.m.) |
| 8. How would you rate the quality of your sleep?  “Sleep Quality” is your sense of whether your sleep was good or poor.  Extremely poor Extremely good  0 1 2 3 4 5 6 7 8 9 10 |
| 9. Comments (if applicable) |

**B) The Core Consensus Sleep Diary in Chinese:**

| 1. 请填写您的被试编号 |
| --- |
| 2. 昨晚关灯上床的时间?  时 分 |
| 3. 从上床到入睡所用的时间?  ________ 分钟 |
| 4. 早上最后一次醒来的时间?  时 分 |
| 5. 早上起床的时间?  时 分 |
| 6. 中间醒了几次?  ________次 |
| 7. 昨晚在床上躺了几个小时?  例：7 /7.5 /8 /8.5 /9小时  ________小时 |
| 8. 昨晚一共睡了几小时?  例：7 /7.5 /8 /8.5 /9小时  ________小时 |
| 9. 对睡眠质量的评分  极其糟糕 极其良好  0 1 2 3 4 5 6 7 8 9 10 |

A diary of nine questions assessing sleep variables such as sleep onset latency (SOL), wake after sleep onset, and sleep quality in its original language (English) and in Chinese.
