# Supplementary material for: Rope skipping or badminton? exercise reduced sleep onset latency in university students
Source: Front Sports Act Living. 2025 May 22;7:1514596. doi: 10.3389/fspor.2025.1514596 (PMC12137340; doi:10.3389/fspor.2025.1514596)
Supplement: Supplementary file 2 [file Table2.docx]

Supplementary Material

# Supplementary Tables

|  |  | *MS* | *F* | *p* | *η^2^* |
| --- | --- | --- | --- | --- | --- |
| Global PSQI score | Time | 84.100 | 28.362 | 0.000*** | 0.381 |
|  | Group | 22.500 | 6.185 | 0.017* | 0.119 |
|  | Group x Time | 1.600 | 0.540 | 0.466 | 0.012 |
| Sleep quality | Time | 4.444 | 20.674 | 0.000*** | 0.310 |
|  | Group | 0.278 | 0.689 | 0.411 | 0.015 |
|  | Group x Time | 0.069 | 0.323 | 0.573 | 0.007 |
| Sleep duration | Time | 0.544 | 2.434 | 0.126 | 0.050 |
|  | Group | 0.100 | 0.202 | 0.655 | 0.004 |
|  | Group x Time | 0.044 | 0.199 | 0.658 | 0.004 |
| Sleep efficiency | Time | 1.600 | 4.488 | 0.040* | 0.089 |
|  | Group | 0.711 | 1.045 | 0.312 | 0.022 |
|  | Group x Time | 0.100 | 0.280 | 0.599 | 0.006 |
| Sleep disturbances | Time | 0.584 | 3.628 | 0.063 | 0.073 |
|  | Group | 1.056 | 3.002 | 0.090 | 0.061 |
|  | Group x Time | 0.251 | 1.557 | 0.218 | 0.033 |
| The use of sleeping medication | Time | 0.017 | 0.107 | 0.745 | 0.002 |
|  | Group | 0.201 | 0.968 | 0.330 | 0.021 |
|  | Group x Time | 0.017 | 0.107 | 0.745 | 0.002 |
| Daytime dysfunction | Time | 5.751 | 15.803 | 0.000*** | 0.256 |
|  | Group | 1.406 | 2.782 | 0.102 | 0.057 |
|  | Group x Time | 1.167 | 3.208 | 0.080 | 0.065 |

**Supplementary Table 2** The table shows the results of the mixed ANOVA test for the other components of sleep of the exercise groups and control group. MS = mean square; PSQI = Pittsburgh Sleep Quality Index. * p < .05, ** p < .01, *** p < .001.
