# Supplementary material for: Rope skipping or badminton? exercise reduced sleep onset latency in university students
Source: Front Sports Act Living. 2025 May 22;7:1514596. doi: 10.3389/fspor.2025.1514596 (PMC12137340; doi:10.3389/fspor.2025.1514596)
Supplement: Supplementary file 3 [file Table3.docx]

Supplementary Material

# Supplementary Tables

|  |  | *MS* | *F* | *p* | *η^2^* |
| --- | --- | --- | --- | --- | --- |
| Global PSQI score | Time | 76.931 | 21.454 | 0.000*** | 0.434 |
|  | Group | 0.754 | 0.253 | 0.619 | 0.009 |
|  | Group x Time | 5.997 | 1.673 | 0.206 | 0.056 |
| Sleep quality | Time | 4.024 | 21.157 | 0.000*** | 0.430 |
|  | Group | 1.178 | 2.771 | 0.107 | 0.090 |
|  | Group x Time | 0.424 | 2.230 | 0.147 | 0.074 |
| Sleep duration | Time | 0.724 | 2.893 | 0.100 | 0.094 |
|  | Group | 2.665 | 5.305 | 0.029* | 0.159 |
|  | Group x Time | 0.391 | 1.562 | 0.222 | 0.053 |
| Sleep efficiency | Time | 0.666 | 1.656 | 0.209 | 0.056 |
|  | Group | 1.883 | 2.528 | 0.123 | 0.083 |
|  | Group x Time | 0.133 | 0.331 | 0.570 | 0.012 |
| Sleep disturbances | Time | 1.015 | 7.251 | 0.012* | 0.206 |
|  | Group | 0.309 | 1.003 | 0.325 | 0.035 |
|  | Group x Time | 0.015 | 0.106 | 0.748 | 0.004 |
| The use of sleeping medication | Time | 0.001 | 0.012 | 0.915 | 0.000 |
|  | Group | 0.068 | 0.648 | 0.428 | 0.023 |
|  | Group x Time | 0.011 | 0.127 | 0.724 | 0.005 |
| Daytime dysfunction | Time | 9.028 | 28.388 | 0.000*** | 0.503 |
|  | Group | 0.068 | 0.127 | 0.724 | 0.005 |
|  | Group x Time | 2.028 | 6.378 | 0.017* | 0.186 |

**Supplementary Table 3.** The table shows the results of the mixed ANOVA for the PSQI scores of the rope-skipping group and the badminton group. MS = mean square; PSQI = Pittsburgh Sleep Quality Index; * p < .05, ** p < .01, *** p < .001.
