# Supplementary material for: Rope skipping or badminton? exercise reduced sleep onset latency in university students
Source: Front Sports Act Living. 2025 May 22;7:1514596. doi: 10.3389/fspor.2025.1514596 (PMC12137340; doi:10.3389/fspor.2025.1514596)
Supplement: Supplementary file 4 [file Table4.docx]

Supplementary Material

# Supplementary Tables

|  |  | *M ± SD* (rope skipping group) | *M ± SD* (badminton group) |
| --- | --- | --- | --- |
| Sleep quality | before | 1.47 ± 0.12 | 1.92 ± 0.14 |
|  | after | 1.12 ± 0.14 | 1.23 ± 1.17 |
| Sleep duration | before | 0.41 ± 0.15 | 1.00 ± 0.17 |
|  | after | 0.35 ± 0.15 | 0.62 ± 0.17 |
| Sleep efficiency | before | 0.65 ± 0.20 | 0.38 ± 0.23 |
|  | after | 0.53 ± 0.17 | 0.08 ± 0.19 |
| Sleep disturbances | before | 1.12 ± 0.11 | 1.23 ± 0.13 |
|  | after | 0.82 ± 0.12 | 1.00 ± 0.13 |
| The use of sleeping medication | before | 0.12 ± 0.07 | 0.08 ± 0.09 |
|  | after | 0.06 ± 0.07 | 0.15 ± 0.09 |
| Daytime dysfunction | before | 2.24 ± 0.15 | 2.54 ± 0.17 |
|  | after | 1.82 ± 0.17 | 1.38 ± 0.19 |
| Global PSQI score | before | 8.06 ± 0.41 | 8.92 ± 0.47 |
|  | after | 6.41 ± 0.46 | 6.00 ± 0.53 |

**Supplementary Table 4.** The table shows the comparisons of the means of other dimensions of sleep of the rope-skipping group and the badminton group.
